# Genetic diversity, population genetic structure and gene flow in the rare and endangered wild plant *Cypripedium macranthos* revealed by genotyping-by-sequencing

**DOI:** 10.1186/s12870-023-04212-z

**Published:** 2023-05-15

**Authors:** Qi Wu, Shang Dong, Yuxin Zhao, Lei Yang, Xiujin Qi, Zhihe Ren, Shubin Dong, Jin Cheng

**Affiliations:** 1 National Engineering Research Center of Tree Breeding and Ecological Restoration, Beijing Key Laboratory of Ornamental Plants Germplasm Innovation and Molecular Breeding, College of Biological Sciences and Technology, Beijing Forestry University, Beijing, China; 2 Department of Yichun, Heilongjiang Academy of Forestry, Yichun, Heilongjiang China; 3 Management Office of Hebei Dahaituo National Nature Reserve, Chicheng, Hebei China

**Keywords:** *Cypripedium macranthos*, Genetic diversity, Population genetic structure, Gene flow

## Abstract

**Background:**

Genetic diversity, genetic structure, and gene flow in plant populations and their influencing factors are important in conservation biology. *Cypripedium macranthos* is one of the few wild orchids with high ornamental value in northern China. However, over the past decade, excessive collection, trading, tourism development, habitat fragmentation, deceptive pollination, and seed germination difficulties have all caused a sharp decline in the number of *C. macranthos* individuals and its population. In order to propose a scientific and effective conservation strategy, the genetic diversity, genetic structure and gene flow of the current CM population are urgent scientific issues to be clarified.

**Results:**

Here, 99 individuals of *C. macranthos* from north and northeast China were analyzed to evaluate the genetic diversity, gene flow among populations, and genetic structure by genotyping-by-sequencing. More than 68.44 Gb high-quality clean reads and 41,154 SNPs were obtained. Our data based on bioinformatics methods revealed that *C. macranthos* has lower genetic diversity, high levels of historical gene flow, and moderate-to-high genetic differentiation between populations. The gene migration model revealed that the direction of gene flow was mainly from northeast populations to north populations in China. The results of genetic structure analysis showed that 11 *C. macranthos* populations can be considered as two groups, and further divided into four subgroups. Moreover, the Mantel test detected no significant “Isolation by Distance” between populations.

**Conclusions:**

Our study demonstrates that the present genetic diversity and genetic structure of *C. macranthos* populations were mainly caused by biological characteristics, human interference, habitat fragmentation, and restricted gene flow. Finally, constructive measures, which can provide a basis for the proposal of conservation strategies, have been suggested.

## Background

The genetic diversity of a species or population is the product of long-term evolution and the premise of its survival and adaptation. The higher the level of genetic diversity of a species, the richer the genetic variation, and the stronger the ability of adapting to different environments. On the one hand, many species may have different levels of genetic diversity and variation to adapt to different environments. On the other hand, habitat fragmentation caused by environmental change or human interference may lead to geographical isolation of natural populations, population degradation, decrease in gene flow, and eventually the loss of genetic diversity and the increase in inbreeding [1]. The difference in spatial genetic structure (SGS) of the population, which refers to the two-dimensional spatial distribution pattern of individual genotypes, is an important embodiment of genetic diversity [2–4]. The formation of SGS is a multi-stage development process affected by factors such as gene flow by pollen or seed dissemination, various natural selection pressures, and the distribution pattern of plants [5]. Plants mainly depend on wind, insects, gravity, and other ways to achieve gene flow by the migration of seeds, pollen, spores, and asexual reproductive organs. Mountains and rivers function as natural geographical barriers affecting gene flow in plants [6–8]. Moreover, human disturbances, such as road or bridge construction and mining, can significantly increase geographical isolation and habitat fragmentation of populations, resulting in reduced gene flow and genetic diversity of populations, thus affecting the genetic structure and population dynamics of the species [9–12]. Even forest park tourism and farming, which have recently become popular in China, pose a threat to rare species.

To formulate scientific and effective conservation strategies and measures for endangered species, it is necessary to understand its genetic diversity, spatial distribution, and relationship with environmental factors, and establish conservation units or select populations with high genetic diversity for in situ or *ex situ* conservation [13–15].

Previous studies of genetic diversity, population genetics, and gene flow mainly used conventional molecular markers (e.g., RAPD, SSR, AFLP, RFLP). However, on the one hand, few loci can be detected by conventional molecular markers, thus, there are some limitations in explaining geographical differentiation and genetic variation in populations, particularly of rare species. On the other hand, developing molecular markers have many steps and is tedious, time-consuming, and laborious. Excitingly, the rapid development of second and third generation sequencing technology, which comes at a lower cost with higher efficiency, provides researchers with more genetic and genomic datasets compared with traditional methods. In addition, a large number of loci that are evenly distributed throughout the genome can be screened. Therefore, using these datasets to analyze population genetic diversity, genetic structure, and gene flow may improve accuracy and resolution [16]. As one of sequencing technology, the genotyping-by-sequencing (GBS) method has been increasingly used in several studies of genetic variation and structure among populations [15, 17–20]. The advantage of the GBS approach is that it not only analyzes the genetic structure and gene flow in non-model species without a reference genome but also provides a basis for interspecific classification, establishing core collections and detecting admixed populations, to discover the fine genetic structure of the population, and particularly can provide unprecedented insight into the genetic diversity, genetic structure, and gene flow in rare and endangered plants [21, 22].

*Cypripedium macranthos* (CM) belongs to the genus *Cypripedium* (Orchidaceae). The global distribution is only in Russia [23], China [24], Japan [25], and the Korean Peninsula [26]. CM has high ornamental value because of its unique petal shape. The labellum of all species in the genus *Cypripedium* has evolved into a pouch shaped like a slipper; thus, these orchids are often called slipper orchids or lady’s slipper orchids [24]. CM is one of the few orchids whose populations in the mainland are mainly distributed in north and northeast China. It thrives in cool climates. Because of the high summer temperature in the north China plain, CM cannot safely spend the summer, so it can only be distributed in some isolated high mountains (More than 1700 m above sea level). Due to high latitude, cool temperatures throughout the year in Northeast China, CM can be distributed from 200 to 1400 m above sea level. Unfortunately, in the past decade, this beautiful wild flower has been excessively and surreptitiously collected for horticultural, medicinal purposes, illegal trade, tourism development, habitat loss, and biological factors (e.g., regeneration difficulties) have pushed this wild species to the brink of extinction [27]. Therefore, CM has been listed as a second-level protected plant in the newly released List of State Key Protected Plants in 2021 in China. In addition to formulating or amending laws and policies at the national level, there is an urgent need to strengthen conservation strategies to avoid further damage to CM. However, little research has been conducted on this beautiful, endangered orchid from mainland China. Thus far, the genetic diversity of CM has not been explored, and research on its genetic structure and gene flow is also absent. Genetic information is the premise and crucial to formulate an effective conservation strategy for this orchid. Although few studies of the genetic diversity of CM or its variants have been conducted using molecular markers abroad [26, 28], most studies of CM in China focus on seed germination [29], morphological and anatomical features [30], reproductive characteristics [31], symbiotic bacteria [32], and seed morphological characteristics and viability [33].

To understand the genetic variation, population structure, and gene flow in CM, and to identify the core population of genetic diversity and propose more accurate conservation strategies, we obtained SNPs of six populations from north China and five populations from northeast China by the GBS method, which can detect a large number of whole genome SNPs. The analysis of SNP characteristics in these different populations provided an insight into the genetic diversity, genetic structure, and gene flow in CM populations under biological barriers (pollinator feeding range and seed germination difficulties) and geographical barriers (Yanshan Mountains and Bohai Sea). Furthermore, we discuss factors that possibly contribute to the genetic diversity and biogeographical pattern of CM populations. Moreover, we assessed the correlation between genetic and geographic distances of CM populations using isolation by distance (IBD) models. Finally, we put forward some suggestions for CM conservation management.

## Results

### SNP screening and genetic diversity

Illumina Hiseq Xten PE150 platform was used to sequenced the remaining 99 samples, and 68.44 Gb of data were obtained, with 468,849,522 reads, average reads per accession was 4,735,853.7, average sequencing depth of all samples was 23.2×, and Q20 value of each sample was above 95.5%. By SNP calling and rigorous screening (software parameter setting: the missing rate < 0.2, minor allele frequency (MAF) ≥ 0.01, read depth (DP) ≥ 4), 41,154 SNPs were obtained from data of all samples. Several main indices of genetic diversity were calculated, and 99 individuals were analyzed, as can be seen from the following indices, the overall level of genetic diversity of CM was low, and the effective number of alleles (Ae) varied from 1.0208 to 2.0000 and average was 1.2342, expected heterozygosity (He) ranged from 0.0204 to 0.5000 with an average of 0.1534, and the extent of observed heterozygosity (Ho) ranged from 0.0000 to 1.0000 and average was 0.1335, the polymorphism information content (PIC) values varied from 0.0202 to 0.3750 with an average of 0.1296, and the nucleotide diversity (Pi) varied from 0.0205 to 0.5031 with an average of 0.1543 (Table 1). 11 populations were compared each other, and the results showed that the Ho, He, PIC, number of alleles (A) and Ae values were the highest in the BHS population (average 0.1658, 0.1749, 0.1465, 1.7427 and 1.2701, respectively), and the lowest in the XAS population (0.1129, 0.0872, 0.0689, 1.2210, and 1.1532, respectively), the data suggested that BHS population had the highest level of genetic diversity among 11 populations and XAS population was the opposite. It is worth noting that our data showed a relatively high genetic diversity of the SLS population although it contained only three individuals. The corresponding genetic diversity indices (Ho, He, PIC, A, and Ae) were 0.1657, 0.1377, 0.1101, 1.3632, and 1.2345 (Table 1). In terms of distribution, the BHS and SLS populations are geographically close to each other, and they may have been one continuous large population historically. Therefore, although the SLS population was subsequently separated from the large population due to habitat fragmentation, it still retains most of the genetic information of the ancestral population.

**Table 1:** The number of individuals and genetic diversity statistics for each population, observed heterozygosity (Ho), expected heterozygosity (He), polymorphism information content (PIC), observed number of alleles, (A) efficient allelic number (Ae), nucleotide diversity (Pi), inbreeding coefficient (Fis), Hardy-Weinberg equilibrium *p* value (HWE-P).

Populations acronym	Distribution area	N	Ho	He	PIC	A	Ae	Pi	Fis	HWE-P
SLS	North China	3	0.1657	0.1377	0.1101	1.3632	1.2345	0.1704	-0.0398	0.9596
YDS_ HTS	North China	6	0.1327	0.1289	0.1056	1.4185	1.2088	0.1421	0.0437	0.9419
BCW	North China	12	0.1179	0.1188	0.0974	1.4233	1.1932	0.1243	0.0422	0.9031
QLL	North China	7	0.1202	0.1152	0.0939	1.3725	1.1887	0.1245	0.0238	0.9373
BHS	North China	17	0.1658	0.1749	0.1465	1.7427	1.2701	0.1805	0.0774	0.8184
YWS	North China	16	0.1227	0.1216	0.1002	1.4768	1.1955	0.1258	0.0308	0.8662
PDS	Northeast China	15	0.1242	0.1264	0.1035	1.4456	1.2060	0.1310	0.0474	0.8849
XAS	Northeast China	5	0.1129	0.0872	0.0689	1.2210	1.1532	0.1191	-0.1284	0.9732
HLHDF	Northeast China	7	0.1407	0.1028	0.0821	1.2948	1.1769	0.1119	-0.2121	0.9554
HLHZ_ HLHC	Northeast China	6	0.1295	0.1172	0.0945	1.3294	1.1966	0.1376	0.0197	0.9559
JSTZB_ JSTZ	Northeast China	5	0.1346	0.1170	0.0941	1.3250	1.1972	0.1315	-0.0544	0.9374
ALL		99	0.1335	0.1534	0.1296	2.0000	1.2342	0.1543	0.0474	0.6006

In the six populations in North China, except SLS population, the inbreeding coefficients (Fis) of all the other populations were positive, and that of BHS population was the highest (Fis = 0.0774). In northeast China, two populations were positive (PDS, HLHZ_HLHC), and three populations were negative (XAS, HLHDF, JSTZB_JSTZ), indicating that inbreeding occurred in most populations in North China. In the Northeast China, both inbreeding and outbreeding occurred.

### Population phylogenetic relationships, principal component analysis, population genetic structure

**Fig. 1:**
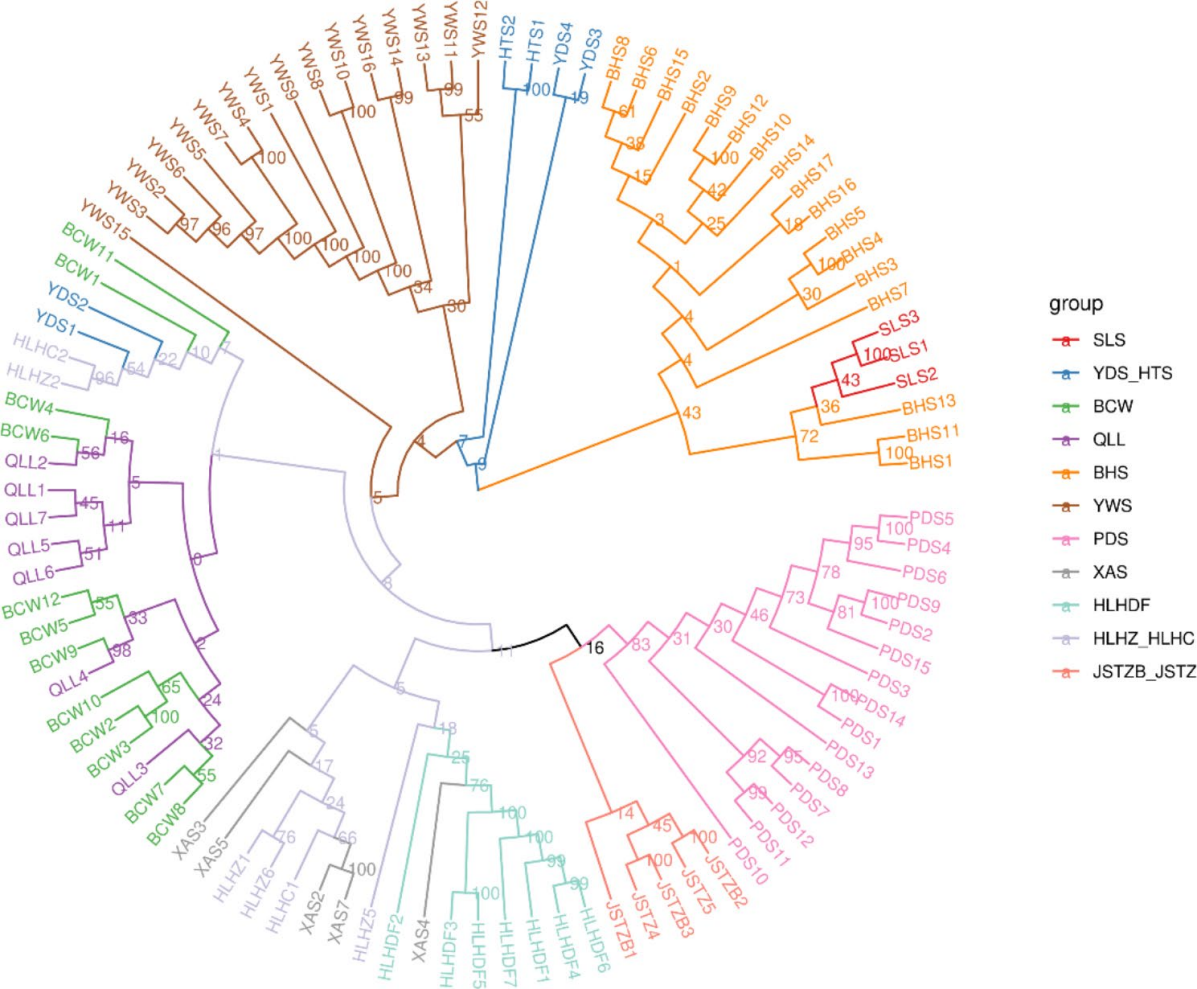
The neighbor-joining phylogenetic tree based on genetic distance matrix representing the grouping of 99 *Cypripedium macranthos* individuals

To understand the relationship between CM populations and visualize their genetic distance, a phylogenetic tree was constructed based on 41,154 SNPs (Fig. 1), and the 99 CM samples were divided into different branches. In the relationships between populations, individuals from the same population clustered together, and clear genetic boundaries were observed between most populations, but some individuals were also interspersed in other branches. First, the PDS, JSTZB_JSTZ, HLHZ_HLHC, HLHDF, and XAS populations were closely related and clustered into a large branch. From the perspective of geographical distribution, these populations were in northeast China; within this large clade, the XAS, HLHZ_HLHC, and HLHDF populations formed a secondary clade with interpopulation infiltration, and these three populations were closely related. Individuals of the SLS population were embedded among the BHS population, and the two populations were clustered into a single branch, indicating a closer relationship between them, this result was consistent with data on genetic diversity we mentioned above. The BCW and QLL populations clustered together and were also geographically close. Surprisingly, two individuals (HLHC2 and HLHZ2) from northeast China clustered with the BCW and QLL populations, which may be related to the prevalence of CM in north and northeast China in the past. Another possibility was that small dust-like seeds of CM were occasionally transported to long distances by strong northeast winds. Sixteen individuals of the YWS population clustered separately. Moreover, the individuals of YDS_HTS population was scattered on different branches, suggesting that YDS_HTS populations may be a mixture of progeny from different ancestral populations.

**Fig. 2:**
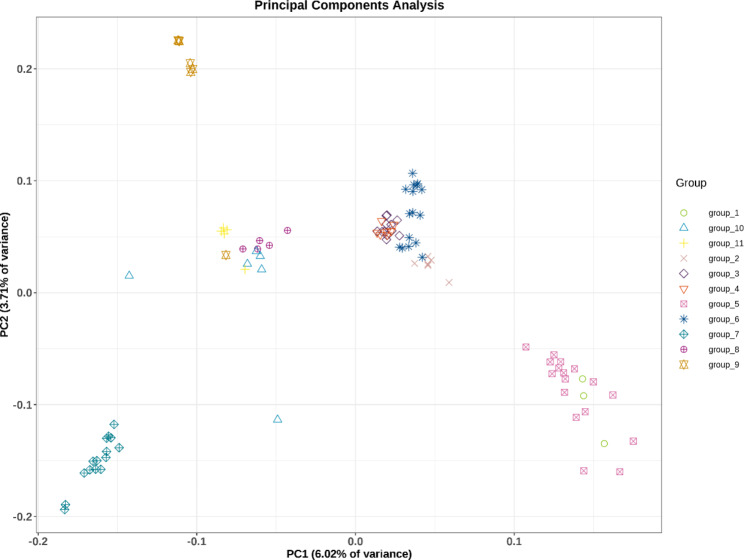
Clustering of *Cypripedium macranthos* populations based on principal components analysis (PCA). Each point represents an individual colored according to the collection site. The six populations from north China are group1–6 (correspond respectively SLS, YDS_HTS, BCW, QLL, BHS and YWS population), the five populations from northeast China are group7–11 (correspond respectively PDS, XAS, HLHDF, HLHZ_HLHC and JSTZB_JSTZ population)

To confirm the clustering of CM individuals, principal components analysis (PCA) was performed based on SNPs obtained from all 99 CM individuals (Fig. 2). PCA showed similar results to the phylogenetic tree analysis: all 99 individuals can be considered as two distinct groups. At PC1, all individuals from five populations (PDS, JSTZB_JSTZ, HLHZ_HLHC, HLHDF, and XAS) in northeast China formed the first group, and the other group comprised all individuals of the six remaining populations from north China (SLS, YDS_HTS, BHS, QLL, BCW, and YWS). At PC2, 99 samples were divided into four clusters: the first comprised all individuals of the SLS and BHS populations; the second contained all individuals of the BCW, YWS, QLL, and YDS_HTS populations; the third had all individuals of the XAS, HLHDF, HLHZ_HLHC, and JSTZB_JSTZ populations; and the fourth consisted only of samples from the PDS population. In the PCA diagram, the individuals of some populations were concentrated and overlapped, suggesting a higher similarity of genetic background between these populations. In contrast, individuals in the HLHZ_HLHC and BHS populations showed some degree of discretization, indicating that individuals in these populations were highly heterogeneous.

**Fig. 3:**
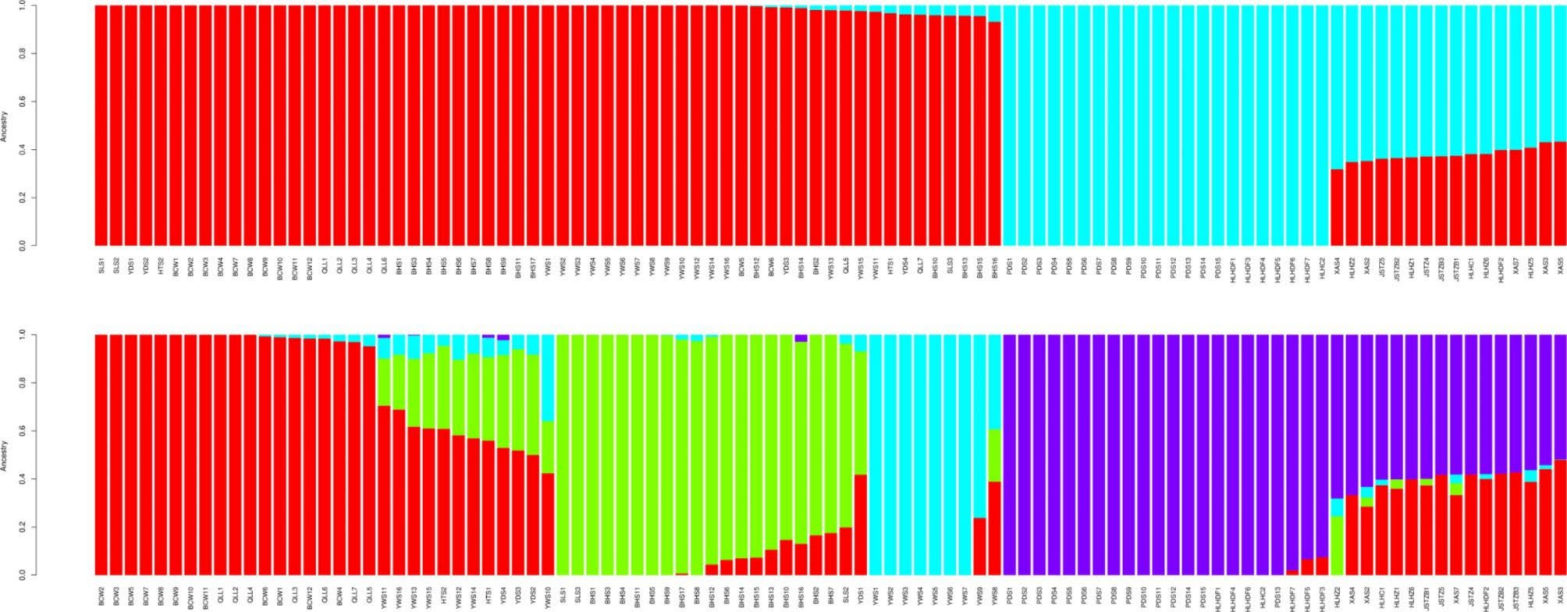
Population structure of 99 *Cypripedium macranthos* individuals from 11 different geographical locations. Population structure analysis suggested that the 99 *Cypripedium macranthos* individuals were divided into 2 group (K = 2) or 4 group (K = 4). Each individual is represented by a vertical bar that is divided by K colored segments representing the likelihood of a membership to each cluster

To complement the PCA and phylogenetic tree analysis and better understand the population structure, an admixture analyses was performed to evaluate the population structure of 99 CM samples (Fig. 3). Usually, the number of clusters into which these samples was divided can be referred to the K value with the lowest cross-validation error (CV), although the lowest CV was observed at K = 4 in this study. Considering the PCA and phylogenetic tree analysis results, we mainly focused on the population structure with K values of 2 and 4. K = 2 indicated that 99 CM samples from 11 populations can be classified into two large clusters (Fig. 3). The first cluster contained samples from all six north China populations (SLS, YDS_HTS, BHS, BCW, QLL, and YWS), and another cluster consisted of five northeast China populations (PDS, XAS, HLHDF, HLHZ_HLHC, and JSTZB_JSTZ), indicating that the populations of north and northeast China had a distinct genetic structure. As K increased to 4, all 99 CM individuals from 11 populations were split into four clusters (Fig. 3). Cluster 1 contained the BCW and QLL populations, 10–16 individuals of the YWS population, and individuals from the YDS_HTS populations. Cluster 2 included all individuals of the BHS and SLS populations, and one sample from the YDS_HTS population. Cluster 3 contained the remaining individuals of the YWS population. Cluster 4, the last cluster, consisted of five northeast China populations. Compared with the population structure when K = 2, the structure of the five northeast China populations did not change, whereas the north China populations split into three groups. Based on the geographical distribution pattern, the north China populations were isolated from each other and were scattered in several high mountains. Thus, substructures were detected in all samples from north China. Even if a few individuals showed a position jump in the phylogenetic tree, some individuals showed that genotype admixtures and substructures exist in the north China populations. Finally we decided the number (K = 2) of genetic clusters that best fit the geographical distribution of CM populations.

### Genetic differentiation and gene flow

Corresponding to the results of the phylogenetic tree and genetic structure analyses, strong gene flow (Nm) and moderate-to-high levels of genetic differentiation were detected between population pairwise comparisons (Table 2). The genetic differentiation (Fst) fluctuated between 0.0031 and 0.2711 in the 11 populations (most pairwise Fst values between populations were greater than 0.1), and with a mean value of 0.1212. The highest Fst value (0.2711) was observed between the SLS and HLHDF populations, and the lowest Fst value (0.0031) was observed between the BCW and QLL populations. According to Wright [33], the genetic differentiation of species was divided into four levels, which are very high (Fst > 0.25), high (0.15–0.25), moderate (0.05–0.15), and almost no differentiation (0.00–0.05). Thus, in the present study, genetic differentiation was evident among most populations, especially between populations from north and northeast China. Similar to genetic differentiation, gene flow (Nm) levels between CM populations were also significantly different. Nm levels ranged from 0.6722 to 80.3951 between different populations (Table 2), and with average 4.0117. Gene flow was higher among populations geographically close to each other, and the highest Nm level (80.3951) occurred between the BCW and QLL populations, and the two populations are located on two mountains 2.9 km apart. The very low level of genetic differentiation and high level of gene flow between the two populations imply that they may have descended from the same population historically. The lowest Nm level (0.6722) occurred between populations SLS of north China and HLHDF of northeast China, because of the biological characteristics of CM (limited seed dispersal distance and low pollinator range). The value of gene flow between populations of northeast and north China indicated historical gene flow.

**Table 2:** Genetic differentiation coefficient and gene flow between different populations. The lower triangle is the interpopulation genetic differentiation coefficient (Fst), and the upper triangle is the interpopulation gene flow

Populations	SLS	YDS_ HTS	BCW	QLL	BHS	YWS	PDS	XAS	HLHDF	HLHZ_ HLHC	JSTZB_JSTZ
SLS	-	2.8025	1.7062	1.8127	6.4345	1.5370	0.9112	1.7516	0.6722	1.7629	1.1474
YDS_HTS	0.0819	-	6.2774	6.0791	11.7692	4.3372	1.3588	2.6039	0.9701	3.1057	1.8333
BCW	0.1278	0.0383	-	80.3951	4.9584	3.5902	1.2615	1.7908	0.9671	2.3788	1.6074
QLL	0.1212	0.0395	0.0031	-	6.0472	3.6870	1.2980	1.8333	0.9232	2.4469	1.6227
BHS	0.0374	0.0208	0.0480	0.0397	-	3.6746	1.4576	4.3625	1.3273	3.9377	2.2626
YWS	0.1399	0.0545	0.0651	0.0635	0.0637	-	1.1043	1.5603	0.8761	1.9624	1.3787
PDS	0.2153	0.1554	0.1654	0.1615	0.1464	0.1846	-	1.6311	0.9292	2.1378	1.5256
XAS	0.1249	0.0876	0.1225	0.1200	0.0542	0.1381	0.1329	-	0.8518	8.4306	2.1447
HLHDF	0.2711	0.2049	0.2054	0.2131	0.1585	0.2220	0.2120	0.2269	-	1.2310	1.0165
HLHZ_HLHC	0.1242	0.0745	0.0951	0.0927	0.0597	0.1130	0.1047	0.0288	0.1688	-	3.1606
JSTZB_JSTZ	0.1789	0.1200	0.1346	0.1335	0.0995	0.1535	0.1408	0.1044	0.1974	0.0733	-

**Fig. 4:**
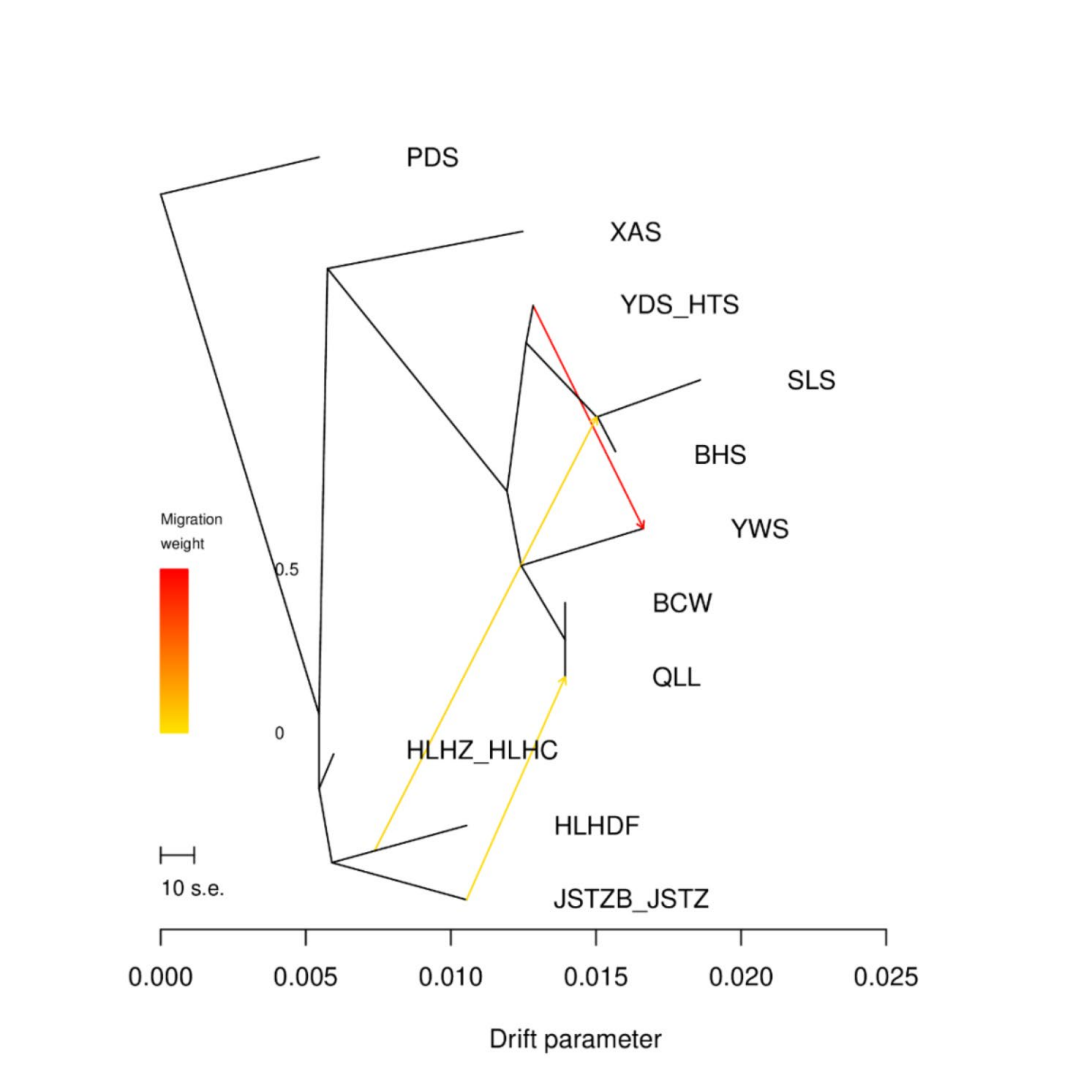
Treemix model analysis showed that gene flow mainly flowed from populations in Northeast China to North China (yellow arrow), but also flowed between populations in North China (red arrow)

Treemix software was used to estimate historical gene flow and determine its direction. The main direction of gene flow was from northeast China populations to north China populations, and between north China populations (Fig. 4). Such gene migration models indicate that the northeast China CM populations may be the ancestors of the north China CM populations, because CM is mainly distributed at high latitudes globally.

### Correlation analysis between genetic and geographic distances

**Fig. 5:**
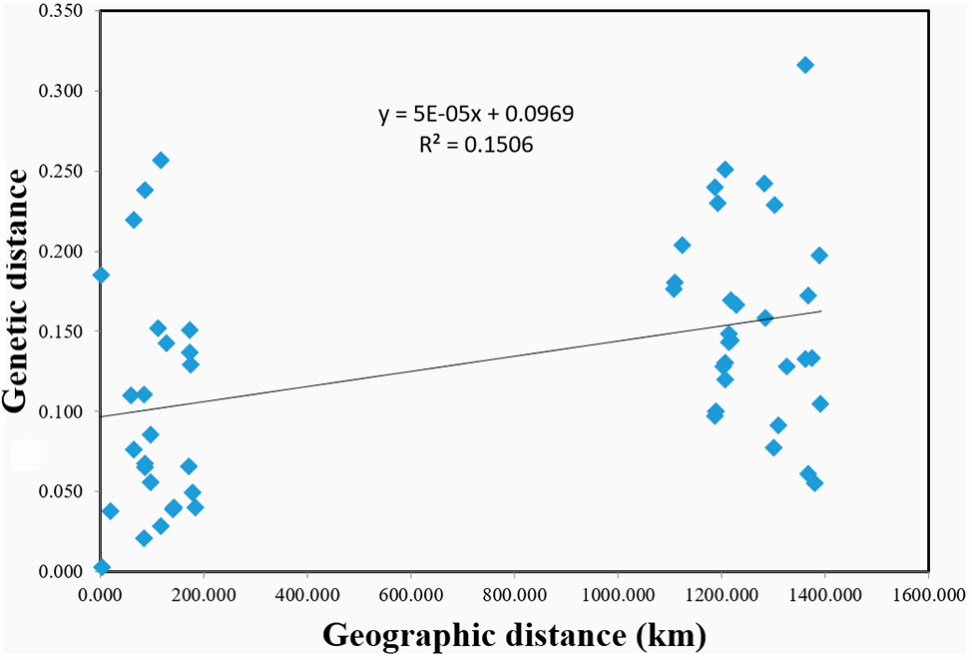
No significant correlations between geographic distance and genetic distance for 11 *Cypripedium macranthos* populations was found through a Mantel test, r = 0.388, P (rxy-rand > = rxy-data) = 0.010. P (rxy-rand > = rxy-data) = probability of positive autocorrelation

A Mantel test was run on all samples to evaluate the effect of geographic distance on population genetic distance. No significant correlation was observed between genetic and geographic distances for the 11 CM populations (r^2^ = 0.1506, P (rxy-rand ≥ rxy-data) = 0.010) (Fig. 5), only 15.06% of genetic divergence were related to geographical distance. Although populations of north and northeast China were separated by more than 1,100 km, a distinct population structure of CM was observed in these two regions (based on PCA and population structure analysis). No IBD model of population was found in this study. This suggested that CM were widely and continuously distributed in north and northeast China and their intermediate zones in history, and that gene flow was frequent among these populations. These existing populations retained most ancestral genetic components; thus, IBD was not obvious. We also performed IBD tests in the region and the results showed that the population of Northeast China (r^2^ = 0.015, P = 0.420), the population of North China (r^2^ = 0.3117, P = 0.010), this indicated that IBD was detected in the North China population to a certain extent, which corresponded to the distribution pattern of the North China population.

## Discussion

Genetic diversity, gene flow, genetic differentiation, population genetic structure, and their influencing factors are the focus of conservation biology research [34]. In particular, studies of rare and endangered species are more relevant and can lay a theoretical foundation for putting forward appropriate suggestions for effective conservation [14, 35–37]. Biological characteristics, historical and contemporary geography and environment, human interference, can all affect the genetic diversity, genetic structure, and gene flow in a species [37, 38]. Gene flow may be the most important factor in the formation of the population genetic structure in insect-borne plants and species with patchy distribution [39, 40]. Another factor is human disturbance, such as habitat fragmentation or degradation, and overharvesting [41, 42]. CM is one of the few orchids with important ornamental and medicinal value in north China. It is mainly distributed in north and northeast China [43], but CM is not found in the southwestern region, which is the diversity center of the genus *Cypripedium*. Thus, CM also has important ecological and scientific value in studying the adaptation and evolution of the genus. Due to human interference, deceptive pollination, and extremely low seed germination rate and other reasons, the number of CM individuals has declined dramatically in recent years. The genetic diversity, genetic structure, and gene flow in CM population need to be urgently studied to put forward appropriate conservation measures. However, most current studies of CM only involve pollination, seed germination, and rhizosphere fungi in China [44]. In this study, we obtained 41,154 SNPs from 99 CM samples using Super-GBS, and the average sequencing depth of the samples was 23.2×. There are studies that have shown that the sequencing depth exceeds 5×, then the subsequent series of analysis results are accurate and reliable [45, 46].

Based on our experimental data, we found some evidence of genetic diversity in CM, several major indices of genetic diversity (Ho and He were 0.1335 and 0.1534, respectively) indicated that the genetic diversity of 99 CM was lower than that of Korea and Japan, Ho and He were 0.140 and 0.185 in Korea and 0.163 and 0.187 in Japan, respectively [26, 28]. Compared with several other species of *Cypripedium*, the level of genetic diversity was significantly lower, such as, *Cypripedium calceolus* (Ho = 0.587, He = 0.572) [47], *Cypripedium flavum* (Ho = 0.431, He = 0.288) [48], *Cypripedium tibeticum* (Ho = 0.664 ± 0.143, He = 0.745 ± 0.119) [49], *Cypripedium kentuckiense* (Ho = 0.514, He = 0.522) [50]. Several factors influence the level of genetic diversity of plants, such as the reproductive characteristics of plants themselves, gene flow, human interference, habitat fragmentation, and chance events. First, although CM reproduces sexually and asexually, its deceptive pollination leads to a low seed setting rate (only 10% or lower), in addition, its seeds have no endosperm and the germination rate is extremely low (less than one in 10,000) [27]. Moreover, asexual reproduction in CM mainly relies on rhizomes, and it is difficult the population to extend to new habitats far away by the asexual reproductive mode, thus reducing the level of genetic diversity of CM. One of the greatest threats to the loss of genetic diversity is a small number of individuals in scattered populations, and this situation is common to rare and endangered species [18]. Second, the pollinators of CM are mainly bumblebees [51], whose feeding and flying range is about 4 km [52], thus, the insect-mediated pollen flow range is limited. Moreover, because of gravity settling and other plant barriers, seed dispersal is shorter. Therefore, the limited gene flow would affect the genetic diversity of CM to some extent. Third, human overharvesting and occasional events (e.g., the Greater Khingan Range forest fire in 1987 that burned an area of 17,000 sq.km) can also lead to the reduction or disappearance of small populations, thereby reducing genetic diversity. There is a unified theory in conservation biology: populations with a large number of individuals tend to be stable and survive longer, whereas those with few individuals may shrink and become extinct, as was observed in this species. For example, five CM individuals were present in Baicaowa Forest Park, Luanping County, north China, in 2013, but our resource survey found that this population disappeared in 2021, the result is the loss of genetic material carried by these individuals. Therefore, although some small populations are not included in nature reserves (e.g., the SLS population, consisting of only three individuals, located near Jinshui Lake), they still need special protection. Otherwise, these small populations may become extinct in a few years.

Each population was analyzed separately, and the BHS population of CM had the highest level of genetic diversity among all populations. This population is located in the Baihuashan National Nature Reserve, which was established in 1985. The number of conserved CM individuals was higher than the other five north China populations. In addition, based on the natural global distribution of CM and its preference for cold climate, the BHS population may be the population that CM spread from north to south and settled down in North China in history, and developed a local CM diversity center. In contrast, the XAS population is located at the Xing’an Temple scenic spot in the center of Yichun city, which is surrounded by the Yichun River and several national roads, forming a geographical barrier. Therefore, the low level of genetic diversity of this population was mainly caused by human interference and the gene flow barrier. Unexpectedly, although SLS population was the smallest population with only three individuals, it was not the lowest in genetic diversity. Considering that this population was located only 21.3 km away from the BHS population, both SLS and BHS populations may have belonged to the same large population historically, SLS populations were split out due to interference from human activities, historical events, and the reproductive characteristics of CM. Thus, it still retains some genetic information of the original large population. Similar results were found not only in the CM population in South Korea [26], but also in *Orchis purpurea*, another plant in the orchid family [53]. In addition, the SLS population is the southernmost population in CM geographical distribution and is close to the scenic spot, which has human disturbance and negative edge effect to some extent; therefore, small populations need more attention and protection because they are also likely to have relatively high genetic diversity.

From the perspective of large-scale geographical scope, the genetic structure analysis, PCA, and phylogenetic tree analysis showed that CM was divided into two groups. The first group consisted of six populations from north China and the second group included five populations from northeast China. These results were consistent with the geographical distribution of CM populations; these populations of north and northeast China were more than 1,000 km apart, separated by the Yanshan Mountains, the Qilaotu Mountains, and the Bohai Sea. On a finer scale, the north China populations were divided into three subgroups. Based on the geographical distribution pattern, these populations were isolated from each other and were scattered in several high mountains. Therefore, substructures were detected in all samples from north China. The distribution pattern of northeast China populations was different. Except for the PDS population, the distribution of most northeast China populations was in plain and hilly areas, and the gene flow barrier was small, especially the individuals of the XAS, HLHZ_HLHC, and JSTZB_JSTZ populations showed obvious genetic admixtures. Thus, the northeast China populations showed up as a whole. Interestingly, 16 samples of the YWS population were divided into different subgroups, suggesting that this population was formed as a result of the intermixing of several ancestors. On the whole, these results of the population structure were similar to those of the PCA and phylogenetic tree analysis, in which although there was some degree of admixture, all the samples still showed a distinct genetic structure.

We calculated genetic differentiation and gene flow among populations, and the levels of genetic differentiation were significantly different among populations, with high genetic differentiation among populations across regions. Based on the classification of genetic differentiation levels by Wright [33], moderate-to-high genetic differentiation (Fst = 0.0542–0.2711) was detected between northeast China populations and north China populations. In this study, the genetic differentiation level of CM was similar to that of the other plants of *Cypripedium* [46]. Although the level of genetic differentiation among populations was high, 11 populations had moderate-to-high gene flow with each other (Nm = 0.6722–80.3951). Gene flow levels varied greatly between populations, with local populations showing high gene flow. According to Wright [33], the intensity of gene flow can be divided into three Nm levels: ≥ 1.0 (strong), 0.250–0.99 (medium), and < 0.249 (low). Thus, genetic differentiation and gene flow between populations were high in this study. How to understand these two seemingly contradictory results? From the perspective of geographical distribution, some of these populations were more than 1,000 km apart. For subalpine and high-latitude plant species, pollen and seed-mediated gene flow were greatly influenced by environmental heterogeneity, and topographic and geomorphic conditions [54, 55]. Gene flow between these populations was almost impossible at present; thus, the detected gene flow was historical gene flow. It can be inferred that CM was widely and continuously distributed in north and northeast China in the past, this speculation is also supported by our study of the MaxEnt species distribution model combined with 38 environmental variables to analyze the suitable geographical range of CM in China in the past and future (The data has not yet been published). All CM can be considered a huge population connected by large gene flow, and the historical gene flow occurred according to the stepping-stone model, short distance gene flow without interference can make continuously distributed population can reach a dynamic balance during a long evolutionary process, but once disturbance occurs, such as excessive collection, environmental change, orogeny in geological history, extreme weather, accidental event and so on, it will accelerate the genetic differentiation and formation of genetic structure of species [13], for example, due to a variety of reasons, forests in Northeast China have been extensively cut down historically, and some woodland has been replaced by farmland, which has had a certain impact on the species in the original habitat under the forest, including CM. Genetic differentiation detected among CM populations is one of evidence that these populations have been subjected to extensive human disturbance and habitat fragmentation in recent times. Various exogenous disturbances can upset this balance and reduce the genetic diversity of species and aggravate genetic differentiation between populations [56]. In addition, the decrease of population size and reproductive fitness will significantly affect gene flow [57, 58]. The current distribution pattern of CM populations is the result of long-term population dispersal in the past and human disturbance in recent times.

We evaluated the direction of gene flow using Treemix software. Our data indicated that the direction of historical gene flow was mainly from northeast China populations to north China populations. It implied that the north China populations were formed by the gradual migration and settlement of northeast populations by seed-mediated gene flow, which is consistent with the dominant distribution of CM and its preference for cold climates. In the resource survey, northeast China populations were widely distributed, ranging from the plain at 200 m above sea level to the Greater Khingan Mountains at more than 1,200 m above sea level, whereas the north China populations were only distributed in the subalpine meadows of several mountains. In addition, the petals of CM individuals in northeast China were purplish red, pure white, and light pink, and individuals with rich color variations in their petals have also been found in the Russian Far East and in Rebun Island, Japan [23, 25]. However, the petals of all CM individuals in north China are purplish red. These phenomena indicate that genetic variation in CM was more abundant at high latitudes and that its diversity center was located at high latitudes. In our comprehensive analysis, these populations from northeast China gradually spread southward in evolutionary history and may be the ancestral populations of the north China populations.

The Mantel test is often used to detect IBD in populations in order to understand the relationship between genetic and geographic distances between populations of different geographical origins [59]. Although the populations of north and northeast China were more than 1,000 km apart, no significant correlation was observed between genetic and geographic distances (Fig. 5). This result may be related to the moderate-to-high historical gene flow that was detected. Strong gene flow counteracted IBD between populations in long evolution history. However, in terms of regional populations, a certain degree of IBD was detected in North China population (r^2^ = 0.3117, P = 0.010), this result corresponds to the pattern of North China populations distributed independently in several high mountains, and inbreeding occurred in most populations. Therefore, more attention should be paid to the population in North China. On the whole, external factors (over-collection, habitat fragmentation, accidental events) and internal factors (deceptive pollination and low seed germination rate) have accelerated the rapid decline in CM population size and distribution area in the past, weakened or even hindered recent gene flow, and finally led to the genetic differentiation of the population. The present distribution pattern and genetic diversity of CM have been formed due to these factors.

## Conclusions

The results of the bioinformatics analyses revealed that CM had a low level of genetic diversity and that genetic differentiation occurred between populations of north and northeast China, showing a distinct genetic structure; in particular, substructures were found among north China populations. These results were mainly due to the comprehensive effects of over-collection, habitat fragmentation, limited recent gene flow, and biological characteristics of CM. Our results provide information on the level of genetic diversity, genetic differentiation, population genetic structure, gene flow, and IBD of CM distributed in mainland China, and provide scientific reference for conservation and rational management measures. To protect the existing population and to re-establish population, we propose the following recommendations: (1) Popularize CM as a national key protected plant and improve awareness of CM protection, especially at forest parks or scenic spots where it is distributed. (2) Strengthen resource surveys and monitor the population size of CM to ensure the accuracy and the integrity of conservation. (3) Collect germplasm resources to establish a germplasm bank for rare and endangered species. Although CM tissue culture was a breakthrough in the laboratory, large-scale tissue culture technology and the survival rate of tissue culture seedlings require to be improved. (4) Gradually reintroduce allogeneic individuals in regions suitable for the growth of CM, such as the middle region of north and northeast China, to build a bridge of gene flow and reduce genetic differentiation between populations. (5) Prioritize protection of north China populations from the perspective of ecological edge effect and island effect. (6) Perform more research on the pollinators of CM and understand the pollination biology of the genus *Cypripedium*, a highly evolved group of plants in the Orchidaceae.

## Materials and methods

### Sample preparation and DNA extraction

In this study, one healthy leaf was collected from each individual as experimental material before June 2021, as CM is mainly propagated by rhizomes, in order to avoid cloning plants, the individuals we sampled were more than 10 m apart, and 106 individuals were randomly selected. The collection was approved by the local forestry departments in Heilongjiang, Hebei and Beijing. The samples were carefully identified by Engineer Shang Dong of Yichun Branch of Heilongjiang Academy of Forestry, a voucher specimen was deposited in the Yichun Forest Museum with an accession number ycl20210607005.

The extraction of DNA from each sample was followed the kit manufacturer’s procedure (TIANGEN BIOTECH (BEIJING) CO., LTD), the quality and concentration of DNA were detected until the requirements for library sequencing were met.

### Library construction and sequencing

The libraries were prepared by following the protocol developed by Qi et al. [60]. Briefly, genomic DNA was digested using MspI and PstI-HF (NEB) at 37℃ for 2 h, then, to inactivate the restriction enzymes at 75℃ for 20 min. The barcoded PstI-HF adapter and common MspI adapater were respectively ligated on the corresponding restriction sites of all samples by T4 DNA ligase (NEB). Ligation reaction lasted for 2 h at 22℃. Following ligation, fragments less than 300 bp were filtered out. PCR amplification was done for each sample separately. PCR products were checked on a 1.0% agarose gel. Primers, dNTP and small DNA fragments were removed from the pooled DNA. Final libraries were sequenced using Illumina Hiseq Xten, PE150 Platform.

### Control the quality of raw reads and SNP calling

In order to ensure the quality of subsequent analysis, Raw Reads were filtered according to the following conditions [60], and fastp (V0.20.0) software [61] was used to control the quality of Raw Reads; removed Reads containing adapter sequences from Raw Reads using the process_radtags program from Stacks (V2.4) (main parameter -r -renz_1 -adapter_mm 1); Clean Reads were obtained by the Fastx_TRIMmer program (main parameter f-1) in fastX Toolkit software package (V0.0.14) to remove the sequence of the restriction site and all bases with the quality score of 3 ‘fastQC less than 20. Since there was no CM genome in the database as a reference, we constructed a GBS reference according to Qi’s method [60], each sample is clustered using the Ustacks program in Stacks (V2.4) software [62], followed by the ASUstacks method to process the clustering results. Blastn software was used to remove tags with similarity greater than 98%, and select tags shared by at least 50% individuals. Follow the above steps to get a GBS reference.

Based on the comparison results between samples and reference, the SNP sites in samples were predicted by GATK software (V3.8-1) Unified program [63]. And used the GATK software SelectVariants program, the preliminary SNP results were obtained by screening the predicted results. To minimize the error rate of SNP detection, VCFtools (V0.1.16) [64] was used to analyze and filter preliminary SNP (The main parameters of program operation are: -- min DP 4; -- MAF 0.01; --max-missing 0.8).

### Genetic diversity analysis

Ho, He, A, Ae, PIC, indexes of CM populations that primarily reflect levels of genetic diversity were analyzed by R package genepop (V1.1.4) as described [65], Pi and the Hardy-Weinberg equilibrium *p* value was calculated VCFtools software (V0.1.16) [64].

### Phylogenetic trees, principal components and population genetic structure analysis

To understand the relationship between populations and visualization of genetic distance, Neighbor-joining tree was constructed with Treebest (V1.9.2) [66] under the p-distances model, with bootstrapping (1,000). Plink2 software (V1.9) [67] was used for PCA analysis of the SNP markers obtained, and the two feature vectors with the greatest influence were obtained. The ADMIXTURE software (V1.3.0) [68] was performed to analyze the genetic structure and degree of admixture among 99 samples, and to determine optimal value of population number (K) with 10-fold cross-validation (CV), then, software Pong (V1.4.7) [69] was used to cluster the repeated results for each K value.

### Genetic differentiation and gene flow analysis

The StAMPP program package (V1.6.1) [70] and Weir and Cockerham’s method [71] was used to calculate the Fst values per pair of CM populations, and the Reynolds’ genetic distance (DR) between populations was estimated by Fst. The gene flow values between CM populations were estimated by formulas (Nm ≈ (1-Fst)/4Fst) [72], and Treemix software (https://speciationgenomics.github.io/Treemix/) [73] was used to evaluate the direction of gene flow among different CM populations.

### Correlation analysis between genetic distance and geographic distance

The Mantel test program in GenAlEx (V6.5) [74] was performed to analyze whether there was a significant correlation between genetic and geographic distances between CM populations, the program runs with 10,000 permutations.

## Data Availability

The raw fastq reads files can be accessed on NCBI Sequence Read Archive (SRA), BioProject’s metadata is available at: https://www.ncbi.nlm.nih.gov/bioproject/PRJNA789327.

## Abbreviations

SNP: Single nucleotide polymorphism
HO: Observed heterozygosity
A: Number of alleles
Ae: Effective number of alleles
HE: Expected heterozygosities
PCA: Principal components analyses
PIC: Polymorphism information content
MAF: Minor allele frequency
GBS: Genotyping-by-sequencing
